# The Advances and Challenges in Utilizing Exosomes for Delivering Cancer Therapeutics

**DOI:** 10.3389/fphar.2018.00735

**Published:** 2018-07-16

**Authors:** Mengliu Yang, Sherry Y. Wu

**Affiliations:** School of Biomedical Sciences, The University of Queensland, Brisbane, QLD, Australia

**Keywords:** exosomes, cancer therapeutics, delivery system, targeted delivery, gene therapy

Nanotechnology plays an important role in advancing treatment and diagnosis of a variety of human diseases. The use of nanocarriers often leads to better pharmacokinetic and safety profiles as well as enhanced bioavailability of the entrapped molecules. Several nanoparticle formulations have already been approved by Food and Drug Administration [Doxil (1995), onivyde (1996), Abraxane (2005)] or have advanced into clinical trials (Andre et al., 2002; Von Hoff et al., 2016; Subbiah et al., 2018). These particles are typically synthesized using lipids or polymers as these materials offer significant protection against degradation from serum nucleases and proteases. Targeting ligands can also be attached to the surface of these systems with ease to enable targeted delivery. However, the reliance of single targeting ligand may not be suitable for treatment of cancer as cancer cells could quickly adapt and change surface receptor expression profiles (Wu et al., 2014). To overcome this problem, recent research has focused on using naturally occurring exosomes to deliver therapeutic cargos. Exosomes, being natural transporters, offer significant advantage for cancer treatment as the surface of exosomes are decorated with numerous ligands that can be beneficial for preferential tumor targeting. Expression of selected ligands can also be enriched through molecular engineering (Alvarez-Erviti et al., 2011).

Certain types of exosomes have been demonstrated to have higher drug delivery efficiency when compared to commonly used nanocarriers (Kim et al., 2016), thus making them ideal candidates for delivering cancer therapeutics. Due to their favorable characteristics including superior targeting capability and safety profile, they are now being investigated as an emerging class of cancer therapeutics in several clinical trials with two trials already entering phase II testing (Besse et al., 2016) (NCT01854866). For instance, dendritic cell (DC)-derived exosomes loaded with tumor antigens have been used to vaccinate cancer patients with the goal of enhancing anti-tumor immune response (Escudier et al., 2005; Morse et al., 2005; Besse et al., 2016). While enhancing T cell response is yet to be achieved in these clinical studies, significant improvement in NK cell activity was observed (Morse et al., 2005; Besse et al., 2016). In addition to targeting immune cells, exosomes have also been used to target tumor cells directly (Kamerkar et al., 2017). The ideal delivery characteristic of exosomes is, in part, due to their surface protein expression profile (e.g., CD47), which allows evasion from phagocytosis by circulating monocytes (Kim et al., 2012; Kaur et al., 2014; Kamerkar et al., 2017). As the result, exosomal nanoparticles have increased circulatory half-life that is beneficial for tumor-targeting (Kim et al., 2016). Despite the promise, the development of exosomal delivery system is still in its infancy, with three major problems requiring further investigation: (1) isolation and purification of exosomes, (2) drug and antigen loading into exosomes, and (3) delivery of cargos to target cells. Here, we discuss recent progress in overcoming these challenges (Figure 1).

**Figure 1:**
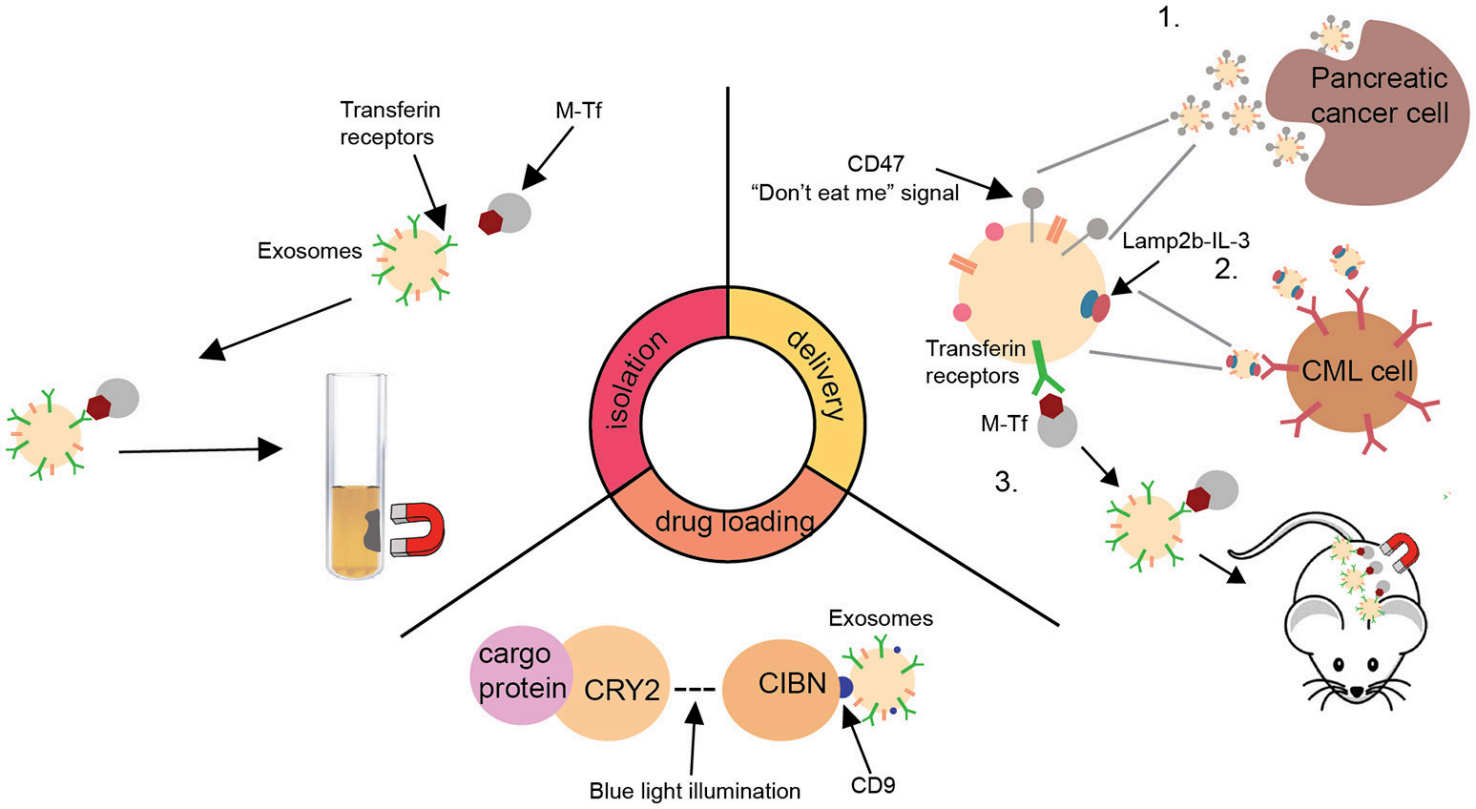
A schematic summary of recent advances in the development of exosome delivery system. Isolation: New exosome isolation method utilizing transferrin-coated superparamagnetic (M-Tf) nanoparticles (Qi et al., 2016). Drug loading: A optically reversible protein-protein interaction (EXPLORs) technology to load proteins into exosomes (Yim et al., 2016). Delivery: Summary of three recent advances in facilitating the delivery of exosomes to tumors (Qi et al., 2016; Bellavia et al., 2017; Kamerkar et al., 2017). CML, chronic myeloid leukaemia; CRY2, cryptochrome 2; CIBN, truncated cryptochrome-interacting basic-helix-loop-helix1; Lamp2b, lysosome-associated membrane protein 2; IL-3, interleukin 3.

Lack of an efficient standardized isolation and purification method is a major challenge for bringing exosome technology into the clinic. It has been reported that exosomes can be isolated and purified by single or combinations of different methods, including immunoaffinity capture, size exclusion, polymeric precipitation, ultracentrifugation, microfluidics techniques, and commercially available kits (Kim et al., 2016; Yim et al., 2016; Bellavia et al., 2017; Kamerkar et al., 2017). Combination of ultrafiltration and ultracentrifugation techniques has been used to generate clinical grade exosomes (Lamparski et al., 2002). An example of this preparation procedure includes concentrating exosomes by ultrafiltrating DC culture media or ascites fluid, followed by ultracentrifugation onto a sucrose/D_2_O density cushion to eliminate non-exosome proteins and to further reduce sample volume (Escudier et al., 2005; Morse et al., 2005; Dai et al., 2008; Besse et al., 2016). This isolation method resulted in generation of exosomes that can be safely administered into patients with minimal toxicity (Escudier et al., 2005). However, due to the complexity and labor intensiveness of this isolation strategy, new methods have been developed to further facilitate future clinical development of exosomal nanosystem. A recent study has described a unique approach to improve the efficiency of exosome isolation procedure. The researchers successfully isolated large number of transferrin receptor-expressing exosomes from reticulocytes, by incubating fresh serum with transferrin-coated superparamagnetic nanoparticles and separating exosomes by magnetic adhesion (Qi et al., 2016). As these exosomes exhibit superparamagnetic behavior with a strong response to an external magnetic field, they can be efficiently separated from the blood. This technology, combined with recently developed nanoscale flow cytometry (Morales-Kastresana et al., 2017), could facilitate the isolation of purified exosomes in large scale and be applied to other ligand of interest. Purification can be achieved through staining the exosomes using Carboxyfluorescein Succinimidyl Ester (CFSE) or other fluorophores followed by size exclusion chromatography and nanoFACS analysis. NanoFACS offers multi-parametric scattered light and fluorescence imaging of exosomes with high resolution and high sensitivity. Its use permits efficient assessment and enhancement of exosome purity.

In addition to developing reliable isolation and purification methods, researchers have recently developed new strategies to load cargos into exosomes. A decade ago, two independent research groups described exosomes as carriers of information and demonstrated their ability to transfer information from one cell type to another (Ratajczak et al., 2006; Valadi et al., 2007). This fundamental concept prompted many researchers to investigate the use of exosomes as a delivery system. Currently, three major types of drug loading strategies have been investigated: incubation (Qi et al., 2016; Bellavia et al., 2017), electroporation (Kamerkar et al., 2017), and sonication (Kim et al., 2016). The most widely used technique for generating cargo-containing exosomes for clinical testing is incubation. For instance, loading of antigens into exosome can be achieved through incubating antigens directly with conditioned DC-culturing media (the source of exosomes) or purified exosomes isolated from the culture media (Escudier et al., 2005; Morse et al., 2005; Besse et al., 2016). While this is a convenient method to load antigen or drug of interest into exosomes, it is hard to precisely control loading efficiency. A recent study has compared three methods of loading, namely incubation, electroporation, and sonication, using paclitaxel as a model molecule. It was shown that a loading efficiency of 29% could be achieved with the sonication approach while 1.5 and 5.3% were achieved for the incubation and electroporation methods, respectively (Kim et al., 2016). However, it must be noted that the sonication method resulted in slight particle aggregation. Thus, development of strategies to overcome the aggregation problem along with further improvement in loading efficiency are critical for future development of exosomal nanotechnology. In addition to traditional methods of drug loading, Yim and colleagues have recently reported a novel loading approach utilizing optically reversible protein-protein interaction (EXPLORs) technology (Yim et al., 2016). The researchers conjugated cargo proteins, mCherry, to photoreceptor cryptochrome 2 (CRY2) and induced their uptake into exosomes by overexpressing tetraspanin protein CD9 conjugated CRY-interacting basic-helix-loophelix1 (CIB1) in exosomes. The interaction between CRY2 and CIB1 was facilitated by blue light illumination. Application of this technology for nucleic acids loading along with strategies to enhance the stability of the resultant particles *in vivo* would be an exciting next set of challenges.

Another area of intense research is the development of better methods to enhance targeting ability of exosomal nanoparticles for cancer treatment. By utilizing normal human foreskin fibroblast-derived exosomes, Kamerkar and colleagues recently demonstrated the ability of exosomes to efficiently deliver Kras^G12D^ siRNA to target undruggable oncogenic Kras in pancreatic tumor cells *in vivo* (Kamerkar et al., 2017). This resulted in diminished oncogenic Kras^G12D^ expression, suppression of cancer cell proliferation, and an increase in overall survival in a mouse model of pancreatic cancer (Kamerkar et al., 2017). It was shown that fibroblast-derived exosomes display favorable protein expression profile on their surface which enabled efficient tumor targeting. These exosomal particles could then be taken up by tumor cells via Ras-induced micropinocytosis. In addition to the presence of naturally occurring ligands on exosomes surface, other researchers have also molecularly engineered exosome-producing cells to enrich the presence of particular ligand(s) on exosome surface in order to target a specific cancer type. For instance, Lamp2b-IL-3 expressing exosome was developed to target chronic myeloid leukemia (CML) cells preferentially as they overexpress IL-3 receptors (Bellavia et al., 2017). Utilizing this molecularly engineered system, Bellavia and colleagues successfully delivered BCR-ABL siRNA to CML cells, making them more sensitive to imatinib therapy in a CML mouse model. This technology could be applied for treatment of other IL-3 receptor over-expressing cancer types, such as lymphoma and acute myeloid leukemia. In addition to molecular methods to enhance targeting ability of exosomes to metastatic tumors, mechanical methods utilizing superparamagnetic nanoparticle-entrapped exosomes in combination with magnetic field at the tumor sites have also been developed to enhance tumor targeting. Using these superparamagnetic exosomes, Qi and colleagues successfully delivered doxorubicin to suppress tumor growth in a subcutaneous mouse model of liver cancer (Qi et al., 2016). This unique technique has provided a new approach to enhance the targeting ability of exosomes to localized tumors.

In summary, recent research has made significant progress in overcoming major barriers for using exosomes as a delivery system. Exosomes are ideal systems for delivering cancer therapeutics, owing to their size, surface expression profiles, low immunogenicity, low cytotoxicity, and long-term safety. Their use has opened a new promising avenue for cancer treatment. Scaling up the production of highly targetable therapeutic exosomes that can be used off-the-shelf which does not require generation from autologous source will be the next critical challenge to bring this promising delivery technology into the clinic.

## Author contributions

SW initial conceptualization of the article. MY and SW wrote the manuscript.

### Conflict of interest statement

The authors declare that the research was conducted in the absence of any commercial or financial relationships that could be construed as a potential conflict of interest.
